# Symptomatic Illness and Low CD4 Cell Count at HIV Seroconversion as Markers of Severe Primary HIV Infection

**DOI:** 10.1371/journal.pone.0078642

**Published:** 2013-11-14

**Authors:** Sara Lodi, Martin Fisher, Andrew Phillips, Andrea De Luca, Jade Ghosn, Ruslan Malyuta, Robert Zangerle, Santiago Moreno, Philippe Vanhems, Faroudy Boufassa, Marguerite Guiguet, Kholoud Porter, for CASCADE Collaboration in EuroCoord

**Affiliations:** 1 Instituto de Salud Carlos III, Madrid, Spain; 2 Brighton and Sussex University Hospitals National Health Service Trust, Brighton, United Kingdom; 3 University College of London, London, United Kingdom; 4 University Division of Infectious Diseases, University Hospital of Siena, Siena, Italy; 5 Université Paris Descartes, EA 3620, Paris, France; 6 Perinatal Prevention of AIDS Initiative, Odessa, The Ukraine; 7 Innsbruck Medical University, Innsbruck, Austria; 8 Hospital Ramon y Cajal, Madrid, Spain; 9 Edouard Herriot Hospital, Lyon, and Universite' de Lyon 1, Lyon, France; 10 Inserm, CESP Centre for Research in Epidemiology and Population Health, U1018, Epidemiology of HIV and STI Team, Le Kremlin-Bicetre, France; 11 INSERM and UPMC Univ Paris 06, UMR S 943, Paris, France; 12 MRC Clinical Trials Unit at University College London, London, United Kingdom; University Hospital Zurich, Switzerland

## Abstract

**Background:**

The risk/benefit of initiating ART in primary HIV infection (PHI) is unclear. The benefits are more likely to outweigh the risks in patients with severe PHI. An accepted definition of severe PHI is, however, lacking.

**Methods:**

CASCADE patients with HIV test interval <6 months were classified as severe and non-severe PHI based on whether the following traits were recorded in the first 6 months following seroconversion: severe specific pre-defined symptoms, central nervous system-implicated illness, and ≥1, ≥2 CD4<350 (and <500) cells/mm^3^. For each definition, we used Kaplan-Meier curves and Cox survival models to compare time to AIDS/death, censoring at the earlier of last clinic visit or 1/1/1997, when combination antiretroviral therapy (cART) became available.

**Results:**

Among 1108 included patients mostly males (85%) infected through sex between men (71%), 366 were diagnosed with AIDS/died. The risk of AIDS/death was significantly higher for individuals with severe symptoms, those with ≥1 CD4<350 cells/mm^3^ or ≥2 CD4 <500 cells/mm^3^ in the first 6 months [aHR (95% confidence interval) 2.1 (1.4,3.2), 2.0 (1.5,2.7), and 2.3, (1.5–3.5) respectively]. Median [interquantile range] survival for patients with ≥2, ≥1 and no CD4<350 cells/mm^3^ within 6 months of seroconversion was 3.9 [2.7,6.5], 5.4 [4.5,8.4] and 8.1 [4.3,10.3] years, respectively. The diagnosis of CNS-implicated symptoms was rare and did not appear to be prognostic.

**Conclusion:**

One CD4 count <350 or two <500 cells/mm^3^ within 6 months of seroconversion and/or severe illness in PHI may be useful early indicators of individuals at high risk of disease progression.

## Background

Although the virological and clinical features of primary HIV infection (PHI) were described nearly 20 years ago [1], [2], it has not been clearly established whether and what characteristics of that early phase are predictive of subsequent faster HIV disease progression. The risk/benefit trade-off of initiating ART in PHI is unclear but the benefits are more likely to outweigh the risks in patients with severe PHI. However, an accepted definition of severe PHI, based on clinical, virological and/or immunological characteristics during that initial period, is lacking.

Several studies have suggested that presence of seroconversion illness with long-lasting symptoms is associated with poorer subsequent outcomes [3], [4], [5]. As some symptoms are more severe than others with symptoms ranging from a simple flu-like symptoms to aseptic meningitis [6], the illness itself and its intensity may be predictive of disease progression. In particular, symptoms which signify the involvement of the central nervous system (CNS), such as meningeocephalitis, are known to be life-threatening, as demonstrated by a recent case of fatal brain necrosis in PHI [7].

Results from observational studies on the predictive value of immunological and virological markers in early HIV infection are inconsistent. Although it has been shown that low levels of CD4 cell count in early HIV infection are predictive of faster disease progression [8], [9], [10], to our knowledge no study has provided a practical definition of what CD4 cell level should be considered as low.

US guidelines for antiretroviral treatment in HIV positive patients indicate cART as optional for patients with ongoing PHI and for those known to have seroconverted in the past 6 months, while European guidelines indicate optional treatment for patients with severe seroconversion illness, although a definition of severity is not provided [11], [12]. The optimal management of patients diagnosed during PHI, however, remains unresolved. We have, therefore, used data from routine clinical practice of patients diagnosed with HIV during, or shortly after HIV seroconversion, to explore definitions of PHI severity based on clinical and immunological features in the first 6 months following HIV seroconversion before the initiation of cART.

## Methods

### Patients

We used data from CASCADE in EuroCoord (www.EuroCoord.net), a collaboration of cohorts of patients with well-estimated dates of HIV seroconversion in Europe, Australia, Canada, and sub-Saharan Africa [13]. The data for this study were pooled in 2006 and, unlike most recent updates, included information on reported seroconversion illness and specific symptoms. The date of HIV seroconversion, used to approximate the date of HIV infection, was estimated by various methods: most frequently as the midpoint between dates of the last negative and first positive HIV antibody test results, the date of laboratory evidence of seroconversion or the date of a seroconversion illness (and an earlier documented negative HIV test result). For this study we restricted to patients with laboratory evidence of seroconversion or an HIV test interval ≤6 months. Patients enrolled in cohorts not reporting information on seroconversion illness and/or with an estimated date of HIV seroconversion after 31/12/1996, when combination antiretroviral therapy (cART) became available, were excluded.

### Ethics statement

All cohorts in CASCADE received approval from their individual ethics review boards. Approval was also given by all ethics review boards to pool anonymised data for analyses and dissemination (see Appendix S1). Signed informed consent was obtained from all patients.

### Definitions of severe primary HIV infection

We explored the following definitions for PHI severity based on clinical characteristics and CD4 measurements within 6 months of HIV diagnosis, i.e. from the first test confirming HIV infection or laboratory evidence of seroconversion, by considering each definition in turn and assigning eligible individuals to whether or not they have the respective trait:

- clinical - severe illness.: symptomatic seroconversion which included at least one of the following disorders judged to be severe *a priori* by a panel of physicians [MF, ADL, RZ SM personal communication]: bronchitis, pneumonia, oral candidiasis, thrombocytopenia, viral meningitis, bacterial meningitis, encephalitis, neuropathy, candida pharyngitis
- clinical - CNS involvement.: symptomatic seroconversion which included at least one of the following disorders indicating neurological involvement agreed by the same panel of physicians: viral meningitis, bacterial meningitis, encephalitis, neuropathy
- immunological - CD4 count <350 cells/mm^3^.: at least one, then at least two CD4 counts <350 cells/mm^3^ within 6 months of HIV seroconversion
- immunological - CD4 count <500 cells/mm^3^.: at least one, then at least two CD4 counts <500 cells/mm^3^ within 6 months of HIV seroconversion
- combinations of clinical and immunological definitions.: severe illness/illness with CNS involvement and at least one CD4 count <350/<500 cells/mm^3^ within 6 months of HIV seroconversion.

Analyses assessing the predictive value of immunological definitions requiring two CD4 cell count measurements were restricted to patients who had at least two CD4 counts measured within 6 months of HIV diagnosis. The cut-points for the immunological definitions were chosen based on current guidelines for treatment initiation in chronic infection [11], [12].

### Statistical analyses

For each of the above definitions, Kaplan-Meier methods were used to compare time to the earlier of AIDS or death for patients with and without each trait. Follow-up was right-censored at the earlier of the last clinic visit or, to avoid selective censoring due to cART initiation in patients with worse prognosis, 31/12/1996.

For each definition, Cox semi-parametric regression models were used to assess the association between each trait and risk of AIDS/death while adjusting for the following potential confounders: sex, age at HIV seroconversion and risk group. For the clinical definitions, we also adjusted for the first recorded CD4 cell count after seroconversion. Potential confounders were defined *a priori* as variables known to be associated with both the specific trait and HIV disease progression, but which are not on the causal pathway. Fractional polynomial models were also fitted to explore the relationship between CD4 cell count predictors and risk of death/AIDS [14]. There was a non-linear age effect on survival and two age groups based on median age at seroconversion (<30 and ≥30 years) were, therefore, used. All models allowed for late entry into the risk set at the time the patient was enrolled into the constituent cohort to minimise survivorship bias. AIDS was defined using the European case definition, i.e. excluding a CD4 count <200 cells/mm^3^ [15]. The prognostic importance of each trait was assessed using Wald tests from the adjusted models and the R^2^ index of explained variation for censored data taking values between 0 and 1 with a higher R^2^ score indicating a stronger association [16]. Statistical analyses were performed with Stata 12.

### Sensitivity analyses

As CD4 count is known to drop transiently at the time of HIV seroconversion, analyses of immunological definitions were also conducted excluding all CD4 counts collected within 4 weeks of the estimated date of seroconversion. Moreover, since patients with missing information on seroconversion illness symptoms or CD4 count within 6 months of HIV diagnosis were excluded from the analyses of the clinical and immunological definitions and this missing data mechanism may not be completely at random, we repeated the analyses imputing the trait for those with missing values [17].

## Results

### Patients

Of the 17146 patients in CASCADE, 1108 were included in the analyses while the remaining patients were excluded as follows: 11083 had an HIV test interval >6 months, 2362 seroconverted after 31/12/1996, 2593 were enrolled in cohorts not reporting information on seroconversion illness. The included patients were mostly males (85%), infected through sex between men (71%) with median [interquantile range (IQR)] first CD4 count of 511 [350,700] cells/mm^3^ recorded after 3.5 months [1.7,14.1] after seroconversion and median age at seroconversion of 29 years [24,35] (Table 1). Clinical progression occurred in 366 patients of whom 326 developed AIDS and 40 died during 4965.6 person-years (PY) of follow-up (incidence rate of AIDS/death 7.4/100 PY). Median [IQR] AIDS-free survival time was 8.0 (4.6,14.0) years. As shown in Table 1, the characteristics of patients included in the clinical and immunological definition analyses were similar to those of the overall eligible patient population. Patients with ≥1 CD4 count had a median [IQR] of 2 [1], [3] CD4 measurements in the first 6 months after HIV diagnosis per patient and a median time from HIV seroconversion to first recorded CD4 count of 2.3 months [0.8,3.7].

**Table 1:** Characteristics of included patients overall and separately for clinical and immunological definitions of severe primary HIV infection (PHI).

	*Overall*	*Clinical definition (known SC illness status)*	*Immunological definition (≥1 CD4 count within 6 months of HIV SC)*
N	1108	865	740
Risk category			
*Sex between men*	789 [71%]	633 [73%]	543 [73%]
*Injecting drug use*	132 [12%]	73 [8%]	34 [9%]
*Sex between men & women*	154 [14%]	126 [15%]	105 [14%]
*Other/unknown*	33 [3%]	33 [3%]	28 [4%]
Sex			
*Female*	166 [15%]	121 [14%]	97 [13%]
Age at SC			
Median [IQR], years	29 [24,35]	30 [25,36]	30 [25,36]
Time first CD4 after SC			
Median [IQR], months	3.5 [1.7,14.1]	3.3 [1.4,11.8]	2.4 [0.8,3.7]
First CD4 after SC			
Median [IQR], cells/mm^3^	511 [350,700]	522 [354,702]	556 [390,740]
HIV test interval			
Median [IQR], days	88 [24,135]	82 [15,132]	76 [14,126]
Year of SC			
Median [IQR], calendar year	1991 [1988,1994]	1991 [1988,1994]	1992 [1989,1994]

Abbreviations: SC = seroconversion, IQR = Interquantile range.

### Clinical definitions

Among 865 patients with information on seroconversion illness, 127 (15%) reported severe symptomatic illness, with Candida pharyngitis (35 cases), oral candidiasis (32), pneumonia (24), bronchitis (23) and viral meningitis (21) being the most common illnesses. Among the 738 patients without severe symptoms, 51% reported a seroconversion illness and the remainder reported no illness. Median [IQR] survival for patients with severe symptoms was 6.3[3.7,-], shorter than the 8.3 [4.8,14.0] years experienced by the remainder (*p* = 0.003, log-rank test). After adjustment for potential confounders, patients with severe illness experienced a hazard of AIDS/death of 2.1 (95% CI 1.4,3.2) compared to those without severe illness (Table 2).

**Table 2:** Risk of AIDS and/or death according to definition of severe primary HIV infection (PHI).

	N	Person-year follow-up	Events	Median [IQR ] survival time, years‡	P-value - unadjusted model¥	Hazard ratio (95% CI)§	P-value - adjusted from Cox model†	R2 (95% CI)
*Clinical definition- severe illness* a								
Yes	127	425.1	41	6.3 [3.7,-]	0.003	2.2 (1.4,3.2)*	<0.001	0.11 (0.06, 0.20)
No	738	3483.1	243	8.3 [4.8,13.9]		1		
*Clinical definition- CNS involvement* b								
Yes	32	120.1	8	- [3.9, -]	0.943	1.0 (0.5,2.2)*	0.932	0.09 (0.05,0.19)
N	833	3788.1	276	8.2 [4.6,14.0]		1		
*Immunological definition- CD4<350 cells/mm^3^*								
Yes	207	680.4	82	5.1 [3.4,7.9]	<0.001	2.0 (1.5,2.7)¤	<0.001	0.10 (0.05,0.19)
No	533	2171.3	139	8.7 [4.8, 11.5]		1		
*Immunological definition- CD4<500 cells/mm^3^*								
Yes	399	1432.5	152	5.5 [3.8,9.1]	<0.001	2.2 (1.6,3.0)¤	<0.001	0.11 (0.6,0.22)
No	341	1419.2	69	10 [5.4,-]		1		
*Immunological definition- 2 CD4<350 cells/mm^3^* ◊								
No CD4 count<350	282	1005.4	77	8.1 [4.3,10.6]	<0.001	1	<0.001	0,08 (0.03,0.22)
One CD4<350	73	260.9	26	5.4 [4.5,8.4]		1.4 (0.9,2.2)		
2 or more CD4<350	88	246.0	38	3.9 [2.7,6.5]		2.5 (1.6,3.9)		
*Immunological definition- 2 CD4<500 cells/mm^3^* ◊								
No CD4 count<500	159	520.4	31	7.5 [4.7,10.0]	<0.001	1	<0.001	0.09 (0.04,0.23)
One CD4<500	95	390.9	32	6.7 [4.9,10.6]		1.1 (0.7,1.9)		
2 or more CD4<500	189	601.0	78	4.8 [3.0,8.4]		2.3 (1.5,3.5)		

a - bronchitis, pneumonia, oral candidiasis, thrombocytopenia, viral meningitis, bacterial meningitis, encephalitis, neuropathy, Candida pharyngitis.

b - viral meningitis, bacterial meningitis, encephalitis, neuropathy.

* Adjusted for sex, risk group (sex between men, injecting drug use, sex between men and women, other/unknown), age at HIV seroconversion (≤30 and >30 years) and first CD4 count (<350, 351–500 and >500 cells/mm^3^).

¤ Adjusted for sex, risk group (sex between men, injecting drug use, sex between men and women, other/unknown), age at HIV seroconversion (≤30 and >30 years).

¥ Long rank test for difference in survivor probabilities.

§ Cox semiparametric survival model.

† Heterogeneity test for categorical variables.

‡ The symbol “-” for median survival time indicates that fewer than 50% of patients at risk have experienced the event by the end of follow-up.

◊ Analyses only included patients with at least 2 CD4 counts measured in the first 6 months of HIV diagnosis.

Seroconversion illness with CNS involvement (21, 19 and 1 cases of viral meningitis, neuropathy and encephalitis, respectively) was reported in 32 patients (3.7%). There was no significant difference in survival between those with and without this trait in univariate or adjusted analyses (Table 2). Among those with and without the trait, 3 (9.4%) and 54 (6.5%) patients, respectively, developed an AIDS event with neurological involvement (dementia, encephalitis, toxoplasmosis or cerebral lymphoma) (p = 0.518, Pearson χ^2^ test).

### Immunological definitions

**Figure 1:**
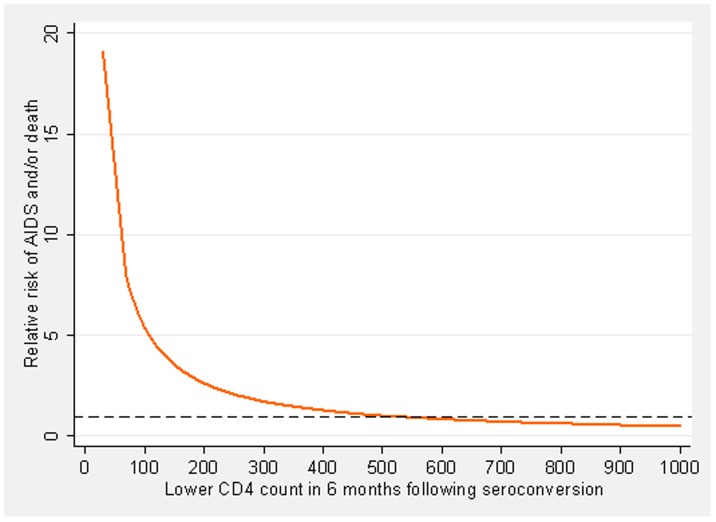
Risk of AIDS and/or death after HIV seroconversion by lowest CD4 count recorded in the first 6 months of HIV diagnosis. The dashed line shows a relative risk of 1 for a CD4 count of 500 cells/mm^3^. Risk adjusted for sex, exposure category and age at HIV seroconversion.

Figure 1 represents the risk of disease progression among 740 patients with ≥1 CD4 count in 6 months of HIV diagnosis as a function of the lowest recorded CD4 count in the first 6 months of HIV diagnosis. The risk of disease progression increased as the lowest CD4 count measured in the first 6 months decreased with a very marked increase in risk for individuals with at least one CD4 count <100 cells/mm^3^.

Among 740 patients with ≥1 CD4 count in 6 months of HIV diagnosis, 207 (28%) and 399 (54%) patients experienced ≥1 CD4 count<350 and ≥1 CD4 count <500 cells/mm^3^, respectively. There was strong evidence of a survival difference between those with and without such a CD4 value for both immunological definitions (p<0.001) in univariable and adjusted analyses (Table 2). The adjusted hazard ratios for those with ≥1 CD4 count <350 and ≥1 CD4 count <500 cells/mm^3^ were 2.0 (95% CI 1.5,2.7) and 2.2 (1.6,3.0), respectively. The R^2^ for the adjusted models for the two definitions suggested that the included covariates explained 10% and 11% of the total variability in clinical progression (Table 2).

443 patients had at least 2 CD4 measurements within 6 months of the HIV diagnosis (180, 263 with two, and three or more measurements, respectively). There was strong evidence of difference in survival for patients with none (n = 282), 1 (n = 73) and ≥2(n = 88) CD4 count <350 cells/mm^3^ within 6 months of HIV diagnosis with median [IQR] survival times of 8.1 [4.3,10.6], 5.4 [4.5,8.4] and 3.9 [2.7,6.5], respectively (p<0.001 log-rank test). Similarly, patients with no, 1 and ≥2CD4<500 cells/mm^3^ had median survival times of 7.5[4.7,10.0], 6.7 [4.9,10.6] and 4.8 [3.0,8.4] years (p<0.001) and proportion alive and AIDS-free at 4 years of HIV seroconversion of 86% (95%CI 77%,92%), 80% (67%,89%) and 60% (50%,68%), respectively.

### Combination of clinical and immunological definitions

Of 604 patients with known seroconversion illness status and at least one CD4 count recorded within 6 months of HIV diagnosis, 31 patients had a severe seroconversion illness and at least one CD4<350 cells/mm^3^. Such individuals were at increased risk of AIDS/death (HR = 2.0, 1.0–3.8) compared to those with a CD4<350 alone (Table 3). Similarly, patients with a severe seroconversion illness and at least one CD4<500 cells/mm^3^ experienced 2.2 (1.3,3.6) times the hazard of clinical progression of patients with a severe illness alone.

**Table 3:** Risk of AIDS and/or death according to combinations of clinical and immunological definitions of severe PHI.

	N	Person-year follow-up	Events	Median [IQR] survival time, years‡	P-value - unadjusted model¥	Hazard ratio (95% CI)§	P-value - adjusted from Cox model†	R2 (95% CI)
*Severe symptoms* a *and/or CD4<350 cells/mm^3^*								
Neither	374	1666.3	100	8.9 [5.0,11.5]	<0.001	0.5 (0.3,0.7)	<0.001	0.11 (0.07,0.23)
CD4<350 alone	140	485.1	57	4.9 [3.7,7.9]		1		
Severe symptoms alone	59	193.7	19	5.8 [4.1,9.3]		1.0 (0.6,1.8)		
Both	31	88.0	13	4.9 [1.4, -]		2.0 (1.0,3.8)		
*Severe symptoms and/or CD4<500 cells/mm^3^*								
Neither	244	1107.1	55	10.0 [5.4,-]	<0.001	0.5 (0.3,0.7)	<0.001	0.11 (0.06,0.22)
CD4<500 alone	270	1044.3	102	6.0 [3.9,9.3]		1		
Severe symptoms alone	33	100.0	5	- [4.3,-]		1.0 (0.3,1.7)		
Both	57	181.6	27	5.4 [2.8,6.3]		2.2 (1.3,3.6)		
*CNS involvement* b *and/or CD4<350 cells/mm^3^*								
Neither	413	1787.8	116	8.7 [4.9,11.5]	<0.001	0.5 (0.4,0.7)	<0.001	0.09 (0.05,0.30)
CD4<350 alone	164	551.2	66	5.1 [3.6,7.9]		1		
CNS involvement alone	20	72.3	3	- [-,-]		0.4 (0.1,1.2)		
Both	7	21.8	4	3.9 [3.4,4.9]		1.9 (0.7,5.5)		
*CNS involvement and/or CD4<500 cells/mm^3^*								
Neither	265	1157.9	60	10 [5.4, -]	<0.001	0.5 (0.3,0.7)	<0.001	012 (0.7,0.23)
CD4<500 alone	312	1181.1	122	5.7 [3.9,9.3]		1		
CNS involvement alone	12	49.3	0	- [-,-]		-		
Both	15	44.8	7	3.9 [2.8,4.9]		1.9 (0.9,4.5)		

a - bronchitis, pneumonia, oral candidiasis, thrombocytopenia, viral meningitis, bacterial meningitis, encephalitis, neuropathy, Candida pharyngitis.

b - viral meningitis, bacterial meningitis, encephalitis, neuropathy.

* Adjusted for sex, risk group (sex between men, injecting drug use, sex between men and women, other/unknown), age at HIV seroconversion (≤30 and >30 years) and first CD4 count (<350, 351–500 and >500 cells/mm^3^).

¤ Adjusted for sex, risk group (sex between men, injecting drug use, sex between men and women, other/unknown), age at HIV seroconversion (≤30 and >30 years).

¥ Long rank test for difference in survivor probabilities.

§ Cox semiparametric survival model.

† Heterogeneity test for categorical variables.

‡ The symbol “-” for median survival time indicates that fewer than 50% of patients at risk have experienced the event by the end of follow-up.

There was no significant difference in survival between patients with a seroconversion illness with CNS involvement and at least one CD4<500 cells/mm^3^ or at least one CD4<350 cells/mm^3^ (Table 3).

### Sensitivity analyses

Excluding CD4 counts in the first 4 weeks of HIV seroconversion or imputing PHI severity status when this was missing did not materially change the results of the analyses (results not shown).

## Discussion

In this large study of patients diagnosed in PHI, we found that patients experiencing any of the following within 6 months of HIV diagnosis were more likely to experience more rapid progression of disease: one CD4 count <350 cells/mm^3^, 2 CD4 counts <500 cells/mm^3^, and/or experience of severe seroconversion illness (any of bronchitis, pneumonia, oral or pharyngeal candidiasis, thrombocytopenia, viral meningitis, bacterial meningitis, encephalitis, neuropathy). We found that seroconversion illness with CNS involvement (ie, viral meningitis, bacterial meningitis, encephalitis, and neuropathy) was rare and there was no statistical evidence to suggest that this associated increased risk of AIDS/death, although confidence intervals were wide.

Our regression models for each definition of severe PHI only explained between 9 and 12% of variability in survival following seroconversion. Thus, these should not be interpreted as prognostic models, since other factors in primary and chronic HIV infection, many of which are unknown, account for the remaining variation. They suggest, however, that in a considerable proportion of patients infected with HIV, progression of HIV disease may already be determined in early infection.

We found that 54% and 28% of patients diagnosed in PHI had at least one CD4 count <500 and <350 cells/mm^3^ measured in the first 6 months, respectively. This is consistent with our previous findings that low CD4 counts are not uncommon in PHI [18]. This study also suggests that early CD4 counts below these levels, especially when recorded more than once, are predictive of fast disease progression. In particular, we found that having 2 CD4<500 cells/mm^3^ in the first 6 months of HIV diagnosis was associated with a probability of being alive and AIDS-free at 4 years of HIV seroconversion of 60% (95% CI 50%,68%), lower than the estimated 86% (77%,92%) for individuals without a CD4<500 cells/mm^3^ at that time. This underlines that immunological status can rapidly deteriorate in the first months following seroconversion in the absence of antiretroviral therapy.

HIV penetrates the CNS very early following infection and severe CNS symptoms have been reported in that period [7], [19]. It has been postulated that neurological involvement at PHI could be an indicator for poor prognosis and subsequent irreversible neurological disease [20], [21]. In our study there was no statistical effect of neurological involvement in PHI on clinical progression of HIV disease and the risk of CNS AIDS events for patients with and without seroconversion illness with CNS involvement was similar. Nevertheless, since the number of patients with seroconversion illness with CNS involvement was small (32 cases), we may have missed a significant effect of this definition of PHI severity.

The prognostic value of HIV-RNA in the first 6 months following seroconversion is controversial and results have shown discrepant conclusion. Whereas the prognostic value of elevated levels of set-point HIV RNA-is well established [22], [23], study investigators have reached discordant conclusions on the predictive value of HIV-RNA levels at the time of seroconversion [2], [8], [9], [24], [25], [26]. Among immunological parameters, low CD8 cell count in early HIV infection have been shown to be correlated with faster disease progression [27], [28]. Unfortunately, we only had limited HIV-RNA data as HIV-RNA in the pre-cART era was not monitored routinely and CD8 cell count is not collected in CASCADE. We were, thus, unable to explore the effect of these markers further. Nevertheless, the advantage of our classification based on clinical and CD4 count in early HIV infection is its relatively simplicity and potential for use in resource limited settings where laboratory infrastructures are lacking.

Our analyses were subject to several important limitations. First, initial studies suggested that long lasting and intense symptoms during PHI are associated with poorer prognosis [3], [4], [5]. It is possible that a better definition of PHI severity would include also number and duration of seroconversion symptoms. As we had no information on the intensity and duration of symptoms we could not explore their predictive values on risk of clinical progression.

Second, reporting of seroconversion illness is subject to recall bias at the time of the HIV-positive test as seroconversion illnesses are more likely to be reported by patients with worst initial prognosis [6]. This would lead to an overestimation of the effect of seroconversion illness on the risk of progression. Nevertheless, we believe that our estimates are unlikely to be affected by self report bias, since severe seroconversion illnesses as defined in this study consisted of clinical symptoms likely to be well-documented in patient records.

Finally, we used data collected in the pre-cART era to avoid selective drop at cART initiation for patients with worse prognosis. Recent studies have reported lower initial CD4 cell counts in individuals who have seroconverted more recently [29] and faster disease progression [30]. It is possible, therefore, that our conclusions about the predictive value of clinical and immunological characteristics are not generalized to more recently-infected individuals.

Initiation of cART in PHI could be beneficial to prevent rapid progression in patients with severe PHI. The best practice for the clinical management of patients diagnosed in PHI or early HIV infection remains unknown and there is ongoing debate on the value, timing and optimal duration of cART in PHI. In three recent randomised controlled trials (RCTs) patients who received transient cART in PHI presented longer times to CD4<350 cells/mm^3^, lower viral set-point or delayed initiation of cART according to recommendations for chronic infection [31], [32], [33] compared with patients who did not receive cART in PHI. Nevertheless, because of the limited follow-up, these trials were unable to answer questions on the beneficial effect of transient early cART in altering long-term disease progression. Moreover, treatment duration in these studies was relatively short, ranging from 12–60 weeks and it is possible that longer durations of transient cART could be more beneficial. No study has examined whether cART initiated in PHI should be continued long-term. The European guidelines recommend that once it is initiated, treatment in PHI should be lifelong [12]. Nevertheless, there is no strong scientific basis to support this statement and unlike treatment in chronic infection, discontinuation of transient cART in PHI has been shown not to be associated with increased HIV morbidity and increases in inflammatory and coagulation markers [34]. Until consensus on the benefit and administration of treatment in PHI is reached, these criteria could be helpful early indicators to identify individuals at risk of rapid HIV disease progression.

In conclusion, this study suggests that one or more CD4<350 cells/mm^3^ and/or severe clinical seroconversion illness, may be a useful indicator for identifying patients who are likely to benefit from early treatment and initial close monitoring. More evidence from RCT is needed to establish the beneficial value of treatment initiation in PHI.

## Supporting Information

Appendix S1 **The CASCADE collaboration in EuroCoord.**
(DOCX)
