# Supplementary material for: Symptomatic Illness and Low CD4 Cell Count at HIV Seroconversion as Markers of Severe Primary HIV Infection
Source: PLoS One. 2013 Nov 14;8(11):e78642. doi: 10.1371/journal.pone.0078642 (PMC3828389; doi:10.1371/journal.pone.0078642)
Supplement: Appendix S1 — The CASCADE collaboration in EuroCoord. (DOCX) [file pone.0078642.s001.docx]

***Appendix S***

**CASCADE Steering Committee**: Julia Del Amo (Chair), Laurence Meyer (Vice Chair), Heiner C. Bucher, Geneviève Chêne, Osamah Hamouda, Deenan Pillay, Maria Prins, Magda Rosinska, Caroline Sabin, Giota Touloumi.

**CASCADE Co-ordinating Centre:** Kholoud Porter (Project Leader), Ashley Olson, Kate Coughlin, Sarah Walker, Abdel Babiker.

**CASCADE Clinical Advisory Board:** Heiner C. Bucher, Andrea De Luca, Martin Fisher, Roberto Muga

**EuroCoord Executive Board**: Heiner Bucher, Basel Institute for Clinical Epidemiology & Biostatistics University Hospital Basel, Switzerland; Fiona Burns, University College London, UK; Geneviève Chêne, University of Bordeaux II, France; Dominique Costagliola, Institut National de la Santé et de la Recherche Médicale, France; Carlo Giaquinto, Fondazione PENTA, Italy; Di Gibb (Scientific Coordinator), Medical Research Council, UK; Jesper Grarup, Københavns Universitet, Denmark; Ole Kirk, Københavns Universitet, Denmark; Jesper Kjaer, Københavns Universitet, Denmark; Laurence Meyer, Institut National de la Santé et de la Recherche Médicale, France; Alex Panteleev, St. Petersburg City AIDS Centre, Russian Federation; Andrew Phillips, University College London, UK, Kholoud Porter, Medical Research Council, UK; Peter Reiss, Academic Medical Center, Netherlands; Claire Thorne (Chair), University College London, UK.

**EuroCoord Council of Partners**: Jean-Pierre Aboulker, Institut National de la Santé et de la Recherche Médicale, France; Jan Albert, Karolinska Institute, Sweden; Silvia Asandi , Romanian Angel Appeal Foundation, Romania; Geneviève Chêne, University of Bordeaux II, France; Dominique Costagliola, INSERM, France; Antonella d’Arminio Monforte, ICoNA Foundation, Italy; Stéphane De Wit, St. Pierre University Hospital, Belgium; Frank De Wolf, Stichting HIV Monitoring, Netherlands; Julia Del Amo, Instituto de Salud Carlos III, Spain; José Gatell, Fundació Privada Clínic per a la Recerca Bíomèdica, Spain; Carlo Giaquinto, Fondazione PENTA, Italy; Osamah Hamouda, Robert Koch Institut, Germany; Igor Karpov, University of Minsk, Belarus; Bruno Ledergerber, University of Zurich, Switzerland; Jens Lundgren, Københavns Universitet, Denmark; Ruslan Malyuta, Perinatal Prevention of AIDS Initiative, Ukraine; Claus Møller, Cadpeople A/S, Denmark; Andrew Phillips, University College London, UK; Kholoud Porter, Medical Research Council at UCL, University College London, United Kingdom; Maria Prins, Academic Medical Centre, Netherlands; Aza Rakhmanova, St. Petersburg City AIDS Centre, Russian Federation; Jürgen Rockstroh (Chair), University of Bonn, Germany; Magda Rosinska, National Institute of Public Health, National Institute of Hygiene, Poland; Claire Thorne, University College London, UK; Giota Touloumi, National and Kapodistrian University of Athens, Greece; Alain Volny Anne, European AIDS Treatment Group, France.

**EuroCoord External Advisory Board**: David Cooper, University of New South Wales, Australia; Nikos Dedes, Positive Voice, Greece; Kevin Fenton, Centers for Disease Control and Prevention, USA; David Pizzuti, Gilead Sciences, USA; Marco Vitoria, World Health Organisation, Switzerland.

**EuroCoord Secretariat:** Kate Coughlin, MRC Clinical Trials Unit in UCL, University College London, UK; Silvia Faggion, Fondazione PENTA, Italy; Lorraine Fradette, MRC Clinical Trials Unit in UCL, University College London,UK; Richard Frost, MRC Regional Centre London, UK; Miriam Sabin, Københavns Universitet, Denmark; Christine Schwimmer, University of Bordeaux II, France; Martin Scott, UCL European Research & Development Office, UK.

**Ethics approval has been granted by the following committees**: Austrian HIV Cohort Study: Ethik-Kommission der Medizinischen Universität Wien, Medizinische Universität Graz – Ethikkommission, Ethikkommission der Medizinischen Universität Innsbruck, Ethikkommission des Landes Oberösterreich, Ethikkommission für das Bundesland Salzburg; PHAEDRA cohort: St Vincent’s Hospital, Human Research Ethics Committee;

Southern Alberta Clinic Cohort: Conjoint Health Research Ethics Board of the Faculties of Medicine, Nursing and Kinesiology, University of Calgary; Aquitaine Cohort: Commission Nationale de l’Informatique et des Libertés; French Hospital Database: Commission nationale de l’informatique et des libertés CNIL; French PRIMO Cohort: Comite Consultatif

de Protection des Personnes dans la Recherché Biomedicale; SEROCO Cohort: Commission Nationale de l’Informatique et des Libertés (CNIL); German HIV-1 Seroconverter Study:

Charité, University Medicine Berlin; AMACS: Bioethics & Deontology Committee of Athens University Medical School and the National Organization of Medicines; Greek Haemophilia Cohort: Bioethics & Deontology Committee of Athens University Medical School and the National Organization of Medicines; ICoNA cohort: San Paolo Hospital Ethic Committee; Italian Seroconversion Study: Comitato etico dell’Istituto Superiore di Sanità; Amsterdam Cohort Studies in Homosexual Men and IDUs: Academic Medical Centre, University of Amsterdam; Oslo and Ulleval Hospital Cohorts: Regional komite for medisinsk forskningsetikk – Øst – Norge (REK 1); Badalona IDU Hospital Cohort: Comité Ético de Investigación Clínica del Hospital Universitari Germans Trias i Pujol; CoRIS-scv: Comité Ético de Investigación Clínica de La Rioja; Madrid Cohort: Ethics Committee of Universidad Miguel Hernandez de Elche; Valencia IDU Cohort: Comité Etico de Investigación Clínica del Hospital Dr Peset-Valencia; Swiss HIV Cohort Study: Kantonale Ethikkommission, spezialisierte Unterkommission Innere Medizin, Ethikkommission beider Basel, Kantonale Ethikkommission Bern, Comité départemental d’éthique de médecine et médecine communautaire, Commission d’éthique de la recherche clinique, Université de Lausanne, Comitato etico cantonale, Ethikkommission des Kantons St Gallen; UK Register of HIV Seroconverters: South Birmingham REC; Early Infection Cohorts: Kenya Medical Research Institute, Kenyatta National Hospital, Uganda Virus Research Institute Science and Ethics Committee, Uganda National Council for Science and Technology, Uganda Virus Research Institute Science and Ethics Committee, Uganda National Council for Science and Technology, University of Zambia Research Ethics Committee, Emory IRB, National Ethics Committee of Rwanda, University of Cape Town Research Ethics Committee, University of Kwazulu Natal Nelson R Mandela School of Medicine; Genital Shedding Study Cohort: University Hospitals of Cleveland, IRB for Human Investigation (CWRU), AIDS Research Committee (ARC), STD/AIDS Control Programme, Uganda Ministry of Health, Committee on Human Research (CHR), Office of Research Administration (UCSF), Biomedical Research & Training Institute (BRTI) – Zimbabwe, Institutional Review Office, Fred Hutchinson Cancer Research Center, Medical Research Council of Zimbabwe (MRCZ).
